# Working conditions and state of health of employees working indoor and outdoor with varying levels of physical demand: Insights from the BIBB/BAuA employment survey 2024

**DOI:** 10.1186/s12889-026-28055-z

**Published:** 2026-06-11

**Authors:** Norman Riedel, Sarah Steghaus, Sascha Wischniewski

**Affiliations:** Federal Institute for Occupational Safety and Health, Dortmund, D-44149 Germany

**Keywords:** Outdoor worker, Job Demands-Resource Model, Musculoskeletal and psychosomatic complaints, Occupational groups, Employment survey

## Abstract

**Background:**

The effects of climate change are posing challenges for occupational health and safety, particularly among outdoor workers performing physically demanding tasks. Drawing on the Job Demands-Resources (JD-R) model, this study systematically examines job demands (physical, environmental, work intensity and working time location), job resources (autonomy and social support) and health outcomes (musculoskeletal and psychosomatic complaints) across four occupational groups: employees working mostly outdoor or indoor with either high or low physical demands. The analyses explore key differences between these groups and identify factors associated with health complaints within them.

**Methods:**

Data from the BIBB/BAuA Employment Survey 2024, a nationally representative dataset comprising approximately 20,000 employees from the German workforce, was analyzed using analyses of variance (ANOVA) and multiple regression models.

**Results:**

Consistent with the JD-R model, findings across all occupational groups highlight the direct associations of working conditions with employee health. Employees in indoor, high-demand roles reported the highest levels of musculoskeletal and psychosomatic complaints, primarily associated with higher work intensity and lower autonomy. Although outdoor, high-demand employees faced stronger environmental and physical demands, they reported fewer health complaints than the indoor, high-demand group.

**Conclusion:**

This absence of a direct association between the environmental demands and health complaints suggests the influence of unmeasured protective factors, such as personal resources. While work-related demands are consistently linked to increased health complaints, job resources, such as autonomy and social support, play a beneficial role in promoting overall well-being. The results highlight the importance of strengthening job resources to safeguard employee health. Enhancing autonomy, social support and personal resources may have beneficial effects that counterbalance work-related demands.

**Supplementary Information:**

The online version contains supplementary material available at 10.1186/s12889-026-28055-z.

## Introduction

The effects of climate change are posing significant challenges for occupational health and safety [1]. Rising temperatures contribute to greater physical fatigue and heat stress, while increased ultraviolet radiation raises the acute risk of eye inflammation and sunburn, as well as long-term risks such as cataracts and skin cancer [2, 3]. The increasing frequency of extreme weather events, including heatwaves, further exacerbates these occupational hazards [4, 5]. These environmental changes particularly affect outdoor workers and those engaged in physically demanding tasks, highlighting the importance of understanding the health impacts in these occupational groups [6]. Occupational heat stress imposes physiological strain, resulting in adverse outcomes such as dehydration, chronic kidney injury and an increased risk of occupational accidents, impairing work capacity and reducing productivity [7–9]. This strain is even greater in physically demanding occupations, which are associated with a higher prevalence of musculoskeletal disorders [10] and an increased likelihood of early work incapacity and disability [11, 12]. The interaction between heat stress and high physical loads is particularly detrimental, correlating with higher rates of workplace accidents [13]. Recent findings from the Health Report of a German Health Insurance Company underscore these disparities [14]: 77% of employees working primarily outdoors reported being affected by climate change at their workplace, compared to 50% of indoor workers. Similarly, employees performing physically demanding work are more likely to be affected than those in sedentary roles (75% vs. 39%). While extreme environmental conditions and high physical demands pose acute risks to workers, such as dehydration or heat stress, the present study focuses on the cumulative, long-term consequences of these working conditions. Chronic exposure to high physical and environmental job demands, especially when compounded by psychological or organizational stressors, often manifests as musculoskeletal and psychosomatic complaints. Specifically, musculoskeletal complaints entail pain, discomfort, or dysfunction within the body’s movement system. Psychosomatic complaints, on the other hand, manifest as physical symptoms, such as tension headaches, sleep disturbances, or gastrointestinal issues, that originate from psychological stressors and cognitive strain.

### Theoretical framework of the study

To systematically investigate the complex interplay between working conditions and health outcomes, this study adopts the Job Demands-Resources (JD-R) model as its theoretical framework [15–17]. According to the model every occupation has unique characteristics, which can be classified into *job demands* and *job resources*. Job demands refer to the physical, psychological, social, or organizational job aspects that require sustained effort and are therefore associated with physiological and psychological costs. Conversely, job resources are the aspects of the job that facilitate achieving work goals, mitigate the impact of job demands, or stimulate personal growth and development. The JD-R model postulates that these categories initiate two distinct psychological processes that influence employee well-being. The first is the health impairment process, which suggests that high job demands lead to exhaustion and health complaints over time. The second is the motivational process, which proposes that the presence of job resources fosters employee engagement and motivation by helping them achieve work goals and reducing the negative impact of demands. The absence of these resources leads to disengagement. The JD-R model proposes that job demands and resources interact rather than functioning in isolation. Specifically, job resources can act as a buffer, reducing the effects of high demands on employee well-being [18]. Additionally, expanded versions of the model include personal resources [19]. These resources work alongside job resources to further prevent exhaustion and increase work engagement. Because of its broad applicability and theoretical flexibility, the JD-R model has been robustly validated across diverse occupational groups, such as nurses, police officers, teachers, and university staff [20–22]. Therefore, it provides an ideal framework for this study.

In the context of the current study, four specific job demands are critical to assessing the health impairment process. First, *physical demands*, such as heavy lifting or prolonged standing, impose significant physiological strain and are associated with a higher prevalence of musculoskeletal disorders [10] as well as an increased likelihood of early work incapacity [11, 12]. Second, *environmental demands* have become increasingly severe due to climate change, where rising temperatures and UV radiation contribute to heat stress, eye inflammation and long-term risks such as skin cancer [2, 3]. Third, *work intensity*, defined as the requirement to work at a high speed or under tight deadlines, serves as a primary psychological stressor [23]. Fourth, *working time location*, referring to the temporal arrangement of work, such as shift work, weekend work or rigid hours, can disrupt circadian rhythms and work-life balance, further exacerbating the strain of physical and environmental demands. Additionally, this study focuses on two key job resources that may buffer these effects through the motivational process. *Autonomy* allows employees control over their tasks and methods, providing a sense of ownership that can mitigate the stress of high demands [24]. *Social support*, derived from colleagues and supervisors is essential for maintaining engagement in challenging work environments [25].

### Objectives

There is a lack of research that systematically examines these job demands, job resources and health outcomes across occupational groups differing in their levels of outdoor work and physical exertion. While qualitative research, such as semi-structured interviews with construction employees, indicates that on-site and office-based employees report differing stressors (e.g., job hazards and limited control versus overtime and job insecurity) [26], a large-scale quantitative comparison is lacking to our knowledge.

The present study aims to fill this gap by leveraging data from the 2024 BIBB/BAuA Employment Survey. This survey provides a large, nationally representative sample of approximately 20,000 employees of the German workforce. Conducted at regular six-year intervals, it offers a comprehensive dataset that simultaneously captures detailed information on job tasks, work requirements, physical and psychosocial working conditions and health complaints, including psychosomatic and musculoskeletal complaints. Based on this data, the study investigates four distinct occupational groups: Outdoor employees with high physical demands (OH), outdoor employees with low physical demands (OL), indoor employees with high physical demands (IH) and indoor employees with low physical demands (IL). This differentiation yields important practical implications for occupational safety and health management. First, by defining distinct stressor-resource-strain profiles for each group, the study offers an assessment of the status quo. This enables the identification of group-specific vulnerabilities, such as the combined impact of environmental and physical demands, that would likely remain hidden in broad workforce analyses. Second, by examining the associations between demands, resources and complaints, the study extends beyond descriptive diagnosis toward intervention-oriented conclusions. It identifies the group-specific demands and resources, thereby offering evidence-based guidance for the development of targeted measures to reduce health complaints. Accordingly, this study addresses two primary research questions:

1. How do the four occupational groups (OH, OL, IH, IL) differ in their job demands, job resources and health complaints?
2. And which of these factors are associated with psychosomatic and musculoskeletal complaints within each group?

## Method

### Study population

The BIBB/BAuA Employment Survey 2024 is a representative cross-sectional survey targeting individuals aged 15 years and older who are engaged in paid employment for a minimum of 10 h per week. As one of the most comprehensive labor market surveys in Germany, it comprises data from 20,006 respondents. Data collection was conducted via computer-assisted telephone interviewing (CATI) with a dual frame sampling (i.e., 50% land line, 50% mobile phone). With a phone number list of the Bundesnetzagentur as a basis, random digit dialing was used. In a first step, not eligible numbers (e.g., invalid phone numbers and non-private households) were excluded. This resulted in a total number of 647,696 phone numbers being left. In a second step, so called unknown eligible participants were excluded (e.g., no one is answering the phone). From the remaining 309,719 participants, 46,190 participants eventually were interviewed (cooperation rate COOP4 of 14.9%). Reasons participants were not included were, for example, that they were not interested, just hung up, or it wasn’t possible to make an appointment within the time frame of the study. Out of all interviews, 43.3% (20006; final sample size) interviewees were aged 15 years or older who are employed. Further exclusion criteria were language barriers, only volunteer work, internships, military and voluntary services or people who were in training. The response rate 2 (RR2) was 7.1%. Further information and a historical overview of the survey can be found in the methodology report [27]. For the present analysis, the unweighted sample was restricted to dependent employees (*n* = 17,474). Our sample may deviate slightly from the exact demographic distribution of the broader workforce, reducing its strict representativeness. However, the primary objective of this study was to investigate the associations between job characteristics and health outcomes rather than to estimate exact population prevalence. The restriction to dependent employees was made since self-employed persons may encounter different forms of demands (such as direct financial and entrepreneurial risks) and resources.

Occupational types were classified into four distinct groups based on two key indicators. The first indicator was derived from a specific survey item that served as a proxy to classify individuals as either predominantly outdoor (O) or indoor (I) workers: *“Do you regularly work outdoors for more than an hour between 10 a.m. and 3 p.m.?”*. Participants answering “Yes” were classified as outdoor workers, participants who answered “No” were classified as indoor workers. The second indicator assessed physical job demands using the item: *“Physical activities that cause breathing and heart rate to increase significantly”*. Individuals who responded with *“frequently”* were classified as having high physical demands (H), while all others (“sometimes”, “rarely”, and “never”) were categorized as having low physical demands (L). Based on this classification, four occupational groups were defined: (a) outdoor workers with high physical demands (OH), (b) outdoor workers with low physical demands (OL), (c) indoor workers with high physical demands (IH), and (d) indoor workers with low physical demands (IL). Within the OH group of the present study’s sample, the most common economic sectors, categorized according to the WZ2008 classification [28], were public administration, defense, and social security (20.0%), followed by the construction industry (12.1%) and health and social services (11.3%). The OL group predominantly comprises sectors such as public administration, defense, social security (14.9%), manufacturing industry (14.2%) and education and teaching (12.5%). The IH group is mainly represented by sectors including health and social services (31.6%), manufacturing industry (19.9%) and trade, maintenance, and repair of motor vehicles (11.9%), while the IL group primarily includes sectors such as manufacturing industry (22.5%), public administration, defense, social security (12.9%) and health and social services (12.0%). Additionally, Table 1 presents descriptive statistics for the study sample, outlining key demographic and employment characteristics for all dependent employees and for each of the four subgroups.

**Table 1:** Sample description

Variables	Type OH	Type OL	Type IH	Type IL	Total
Age	47.4 (12.5)	49.6 (11.4)	50.5 (11.1)	50.0 (11.0)	49.9 (11.1)
Sex					
Male	75.7%	72.7%	47.5%	53.8%	56.3%
Female	24.3%	27.3%	52.5%	46.2%	43.7%
Employment status					
Full-time	83.3%	80.1%	70.3%	74.2%	75.0%
Part-time	16.7%	19.9%	29.7%	25.8%	25.0%
Level of education					
No vocational training	7.0%	4.1%	4.6%	2.9%	3.3%
Vocational training qualification	63.1%	50.6%	61.4%	36.6%	40.6%
Advanced training qualification	7.7%	10.1%	9.4%	8.5%	8.7%
University degree	16.9%	31.7%	21.7%	49.5%	44.7%
Other educational qualification	5.3%	3.5%	2.9%	2.5%	2.7%
Requirement level					
Unskilled/ Semi-skilled	4.2%	4.5%	9.3%	2.6%	3.2%
Skilled	75.5%	52.8%	57.0%	36.5%	41.0%
Complex specialized	11.8%	19.0%	15.0%	21.9%	20.8%
Highly complex	8.5%	23.7%	18.7%	39.0%	35.0%
Weekly working hours	36.7 (7.9)	35.6 (7.9)	33.4 (8.7)	34.3 (8.1)	34.5 (8.2)
Job tenure (in years)	14.5 (12.5)	14.3 (12.0)	15.4 (12.0)	15.3 (12.0)	15.2 (12.0)

For age, weekly working hours and job tenure means and SDs are reported

*OH* Outdoor, high demand, *OL* Outdoor, low demand, *IH* Indoor, high demand, *IL* Indoor, low demand

### Dependent variables

The BIBB/BAuA Employment Survey 2024 includes a comprehensive set of items designed to assess various job demands, resources and health complaints. The selection and categorization of demands and resources were guided in particular by the JD-R model and the Job-Exposure-Matrix (JEM) based on the BIBB/BAuA Employment Survey [29, 30]. The JEM aims to provide a tool to classify and monitor working conditions and describing different occupations in regard to their exposure for different job demands. The scales that assessed job demands include physical and environmental demands, work intensity and demands related to working time location. As job resources, the survey operationalized autonomy and social support at the workplace. In total, 31 items on 6 scales were selected and assigned to the respective demands and resources categories. Furthermore, health complaints were assessed with 19 items and categorized into musculoskeletal and psychosomatic complaints. Brief item descriptions are provided in Table 1 of the Additional File 1.

In the BIBB/BAuA Employment Survey 2024, employees were asked to indicate, on a four-point scale ranging from 1 (frequently) to 4 (never), how often they experienced specific demands and resources at work. For analytical purposes, these items were recoded dichotomously: responses were coded as 1 if the demand/resource occurred frequently and 0 otherwise. Certain items required deviations from this general coding scheme. The item asking whether participants feel over- or underchallenged (F410) within the *work intensity* scale included three response options: (1) usually up to the requirements, (2) rather overchallenged, and (3) rather underchallenged. Here, responses of “rather overchallenged” were coded as 1, and all others as 0. Items in the *working time location* scale featured binary (yes/no) response options. For working at least once a month on Saturdays (F220) and working at least once a month on Sundays (F223), “yes” responses were coded as 1 and “no” as 0. Whereas for item F209 (“Do you normally work between 7 and 19 o’clock?”), the coding was reversed. Health complaints were assessed using binary (yes/no) items, with “yes” responses coded as 1, and “no” as 0.

For each scale, summed scores were calculated by adding the recoded items, resulting in scale values ranging from 0 (no demands/resources/complaints reported) to the maximum number of items per category (all reported). These scales were then included in the statistical analyses. The internal consistency of each scale was evaluated using Cronbach’s α and McDonald’s ω, calculated on the subsample of all dependent employees (see Table 2).

**Table 2:** Cronbach’s alpha and sample statistics

Categories	Cronbach’s α	McDonald’s ω	Mean	SD	*n*
Demands					
Physical demands (4 Items)	0.655	0.668	0.804	1.073	17,530
Environmental demands (6 Items)	0.690	0.701	0.817	1.273	17,532
Work intensity (7 Items)	0.660	0.666	2.348	1.719	17,442
Working time location (3 Items)	0.715	0.764	0.609	0.953	17,556
Resources					
Autonomy (6 Items)	0.597	0.590	3.817	1.547	17,228
Social support (5 Items)	0.633	0.619	3.542	1.328	16,788
Complaints					
Musculoskeletal complaints (8 Items)	0.713	0.710	1.621	1.767	17,519
Psychosomatic complaints (11 Items)	0.747	0.766	2.090	2.169	17,468

*SD* Standard deviation

### Statistical analysis

To address the research questions, variance and regression analyses were employed. All computations were based on the subsample of dependent employees. To examine Research Question 1 (i.e., differences in demands, resources and complaints across occupational groups), analyses of variance (ANOVAs) were conducted for all dependent variables. Since Levene-tests indicated a violation of homogeneity of variances for all dependent variables, Welch-ANOVAs were performed for correction. To control for Type I error, *p*-values from the post-hoc tests were adjusted using Tukey’s HSD procedure. The significance level for all statistical analyses was set a priori at α = 0.05. Effect sizes are reported as eta squares (η^2^). In accordance with standard guidelines [31], η^2^ values between 0.01 and 0.06 indicate a small effect, values between 0.06 and 0.14 indicate a medium effect, and values above 0.14 indicate a large effect.

To investigate Research Question 2 (i.e., the relationship between demands, resources and complaints across occupational groups), multiple linear regression analyses with standardized beta-coefficients and robust standard errors (HC3) were conducted separately for each of the four occupational groups, focusing on psychosomatic and musculoskeletal health complaints. All metric variables were z-standardized prior to inclusion in the regression models. In order to rule out alternative explanations for occupational group effects, several control variables were included in the analyses. Adjustments were made for age and gender, as baseline physiological health and the prevalence of musculoskeletal and psychosomatic complaints often vary systematically across these demographics. To account for the cumulative exposure to job demands, actual weekly working hours and job tenure were included as controls. Furthermore, the analyses were adjusted for educational attainment and job requirement level. These variables serve as important proxies for socioeconomic status and cognitive capacity, by which an employee’s coping mechanisms and overall health can be independently influenced. Finally, employment status was included to control for the structural differences in employment arrangements (full-time versus part-time). All statistical analyses were performed with IBM SPSS Statistics (Version 29, IBM Corp., Armonk, NY, USA).

## Results

### Differences in demands, resources, and complaints across occupational groups

Table 3 reports the means and standard deviations for the demands (physical demands, environmental demands, work intensity, working time location), resources (autonomy, social support) and health complaints (musculoskeletal and psychosomatic complaints). Table 4 presents the F-statistics of the ANOVA models testing differences in mean values across the four occupational groups, along with the corresponding post-hoc results. All analyses revealed significant main effects of the occupational group, with effect sizes ranging from large (physical and environmental demands) to small (work intensity, working time location, autonomy, social support, musculoskeletal and psychosomatic complaints). Overall, the findings suggest systematic variation in working conditions and health complaints across occupational groups.

**Table 3:** Descriptive statistics for all dependent variables across all occupational groups

Variables	Type OH		Type OL		Type IH		Type IL	
	M (SD)	*n*	M (SD)	*n*	M (SD)	*n*	M (SD)	*n*
Demands								
Physical Demands	2.50 (1.18)	671	1.22 (1.15)	1,823	2.17 (1.20)	1,070	0.55 (0.85)	13,855
Environmental Demands	2.59 (1.59)	671	1.36 (1.44)	1,814	2.11 (1.59)	1,069	0.55 (1.02)	13,865
Work Intensity	2.77 (1.81)	668	2.00 (1.61)	1,803	3.37 (1.86)	1,063	2.29 (1.69)	13,793
Working Time Location	0.93 (1.06)	672	0.70 (0.98)	1,818	1.40 (1.13)	1,070	0.51 (0.88)	13,875
Resources								
Autonomy	3.46 (1.62)	665	3.88 (1.63)	1,784	3.27 (1.60)	1,047	3.88 (1.51)	13,620
Social Support	3.37 (1.47)	649	3.52 (1.34)	1,726	3.26 (1.50)	1,028	3.58 (1.30)	13,274
Complaints								
Musculoskeletal Complaints	2.70 (2.17)	674	1.73 (1.80)	1,813	2.90 (2.11)	1,071	1.45 (1.64)	13,845
Psychosomatic Complaints	2.71 (2.44)	671	1.98 (2.15)	1,811	3.32 (2.5)	1.064	1.98 (2.09)	13,812

*M* Mean value, *SD* Standard deviation, *OH* Outdoor, high demand, *OL* Outdoor, low demand, *IH* Indoor, high demand, *IL* Indoor, low demand

**Table 4:** Results of the Welch-ANOVAs for all dependent variables

Variables	F (df1, df2)	*p*	η^2^ [95% CI]	Sign. Post hoc comparisons
Demands				
Physical Demands	1318.90 (3, 1796.43)	< 0.001***	0.257 [0.246; 0.267]	all comparisons
Environmental Demands	822.42 (3, 1776.85)	< 0.001***	0.194 [0.184; 0.204]	all comparisons
Work Intensity	151.34 (3, 1861.62)	< 0.001***	0.029 [0.024; 0.035]	all comparisons
Working Time Location	254.05 (3, 1830.81)	< 0.001***	0.058 [0.052; 0.065]	all comparisons
Resources				
Autonomy	58.99 (3, 1838.40)	< 0.001***	0.011 [0.008; 0.014]	OH-OL, OH-IL, OL-IH and IH-IL
Social Support	18.65 (3, 1783.72)	< 0.001***	0.004 [0.002; 0.006]	OH-IL, OL-IH and IH-IL
Complaints				
Musculoskeletal Complaints	231.68 (3, 1828.15)	< 0.001***	0.055 [0.048; 0.061]	OH-OL, OH-IH, OH-IL, OL-IH and IH-IL
Psychosomatic Complaints	114.10 (3, 1843.64)	< 0.001***	0.025 [0.021; 0.030]	OH-OL, OH-IH, OH-IL, OL-IH and IH-IL

Bold *p* values indicate significance. For post hoc comparisons Tukey test were used

*F* F-statistics, *df* Degrees of freedom, *p* p-value, *CI* Confidence interval, *η*^2^ partial eta-squared, *OH* Outdoor, high demand, *OL* Outdoor, low demand, *IH* Indoor, high demand, *IL* Indoor, low demand

#### Demands

A more detailed examination revealed that all demand categories differ significantly between occupational groups. With respect to *physical* and *environmental demands*, employees in the OH group showed the highest levels, followed by those in the IH group, then the OL group, and finally the IL group. Regarding *work intensity*, the highest levels were observed in the IH group, followed in descending order by the OH, IL, and OL groups. For *working time location*, the most unfavorable conditions were again found in the IH group, followed by the OH group, then the OL group and lastly the IL group.

#### Resources 

The highest levels of *autonomy* were observed among employees in the OL group and the IL group. In contrast, employees in the OH and IH groups exhibited the lowest levels of autonomy. Moreover, *social support* was more pronounced among employees in the OL and IL groups compared to those in the OH and IH groups.

#### Complaints 

Employees in the IH group reported the highest levels of *musculoskeletal complaints*, followed by those in the OH group, then the OL and IL groups. A similar pattern emerged for *psychosomatic complaints*, with the highest levels observed among employees in the IH group, followed by the OH group. Employees in the OL and IL groups reported the lowest psychosomatic complaints.

### Variables associated with complaints across occupational groups.

**Fig. 1:**
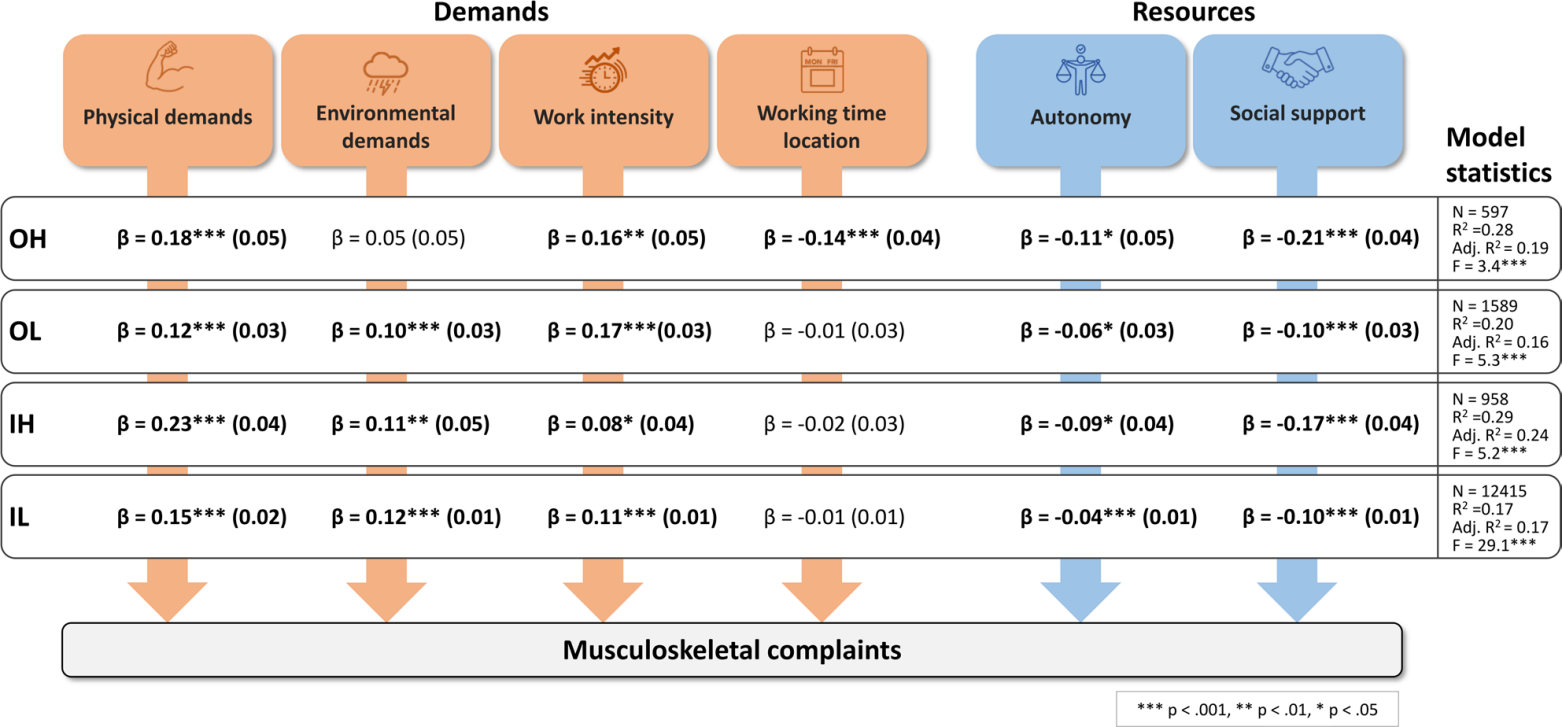
Regression models examining the associations of job demands and resources with musculoskeletal complaints across four occupational groups (OH, OL, IH, IL). Values presented are standardized beta coefficients (β) with heteroscedasticity-consistent standard errors (HC3) in parentheses. Bold values indicate significance. All models were adjusted for age, gender, weekly working hours, job tenure, educational attainment, employment status, and job requirement level. Model statistics are provided on the right

**Fig. 2:**
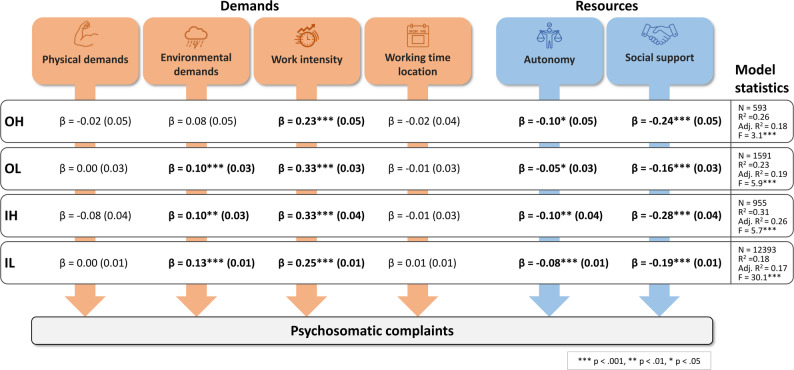
Regression models examining the associations of job demands and resources with musculoskeletal complaints across four occupational groups (OH, OL, IH, IL). Values presented are standardized beta coefficients (β) with heteroscedasticity-consistent standard errors (HC3) in parentheses. Bold values indicate significance. All models were adjusted for age, gender, weekly working hours, job tenure, educational attainment, employment status, and job requirement level. Model statistics are provided on the right

Figure 1 presents the results of the regression analyses examining the relationship between working conditions and musculoskeletal complaints, while Fig. 2 shows the results for the relationship between working conditions and psychosomatic complaints.

#### Musculoskeletal complaints 

Across all occupational groups, higher *physical demands* (β = 0.12 to 0.23) and higher *work intensity* (β = 0.08 to 0.17) were associated with significantly more musculoskeletal complaints. Among employees in the OL, IH and IL groups, higher *environmental demands* (β = 0.10 to 0.12) were also linked to increased musculoskeletal complaints. In contrast, for employees in the OH group, higher environmental burdens did not appear to be associated with increased musculoskeletal complaints. Interestingly, among employees in the OH group, working at weekends and outside the hours of 7 a.m. to 7 p.m. was associated with fewer musculoskeletal complaints (β = − 0.14), whereas *working time location* showed no effect on musculoskeletal complaints in the other occupational groups. Job resources, such as higher *autonomy* (β = − 0.11 to − 0.04) and higher levels of *social support* (β = − 0.21 to − 0.10), were associated with fewer musculoskeletal complaints across all occupational groups.

#### Psychosomatic complaints 

*Physical demands* appeared to have no association with psychosomatic complaints across all occupational groups. Higher *environmental demands* (β = 0.10 to 0.13) were associated with more psychosomatic complaints among employees in the OL, IH and IL groups, whereas environmental demands are not linked to psychosomatic complaints in the OH group. Higher *work intensity* (β = 0.23 to 0.33) was linked to significantly more psychosomatic complaints across all occupational groups, while *working time location* showed no association with psychosomatic complaints. Job resources, such as *higher autonomy* (β = − 0.10 to − 0.05) and higher levels of *social support* (β = − 0.28 to − 0.16), were associated with fewer psychosomatic complaints across all groups.

Further, sensitivity analyses were conducted assuming continuous scales rather than the dichotomized variables. The results of this re-analysis demonstrated that the majority of the observed associations remained unchanged in both direction and significance. However, four specific associations now also reached significance. Specifically, the associations between environmental demands and both musculoskeletal and psychosomatic complaints became statistically significant for the OH group. Additionally, the association between physical demands and psychosomatic complaints reached significance for both the IH and IL groups. In this case, the results indicate that higher physical demands are associated with lower psychosomatic complaints. The complete results of the sensitivity analyses are provided in Figures 1 and 2 of the Additional File 1.

## Discussion

This study utilized a large, nationally representative dataset to examine the interplay between job demands, job resources and health complaints across four occupational groups, categorized by work location (indoor vs. outdoor) and physical demands (high vs. low). In the following discussion, the key differences identified between these occupational groups are first addressed (Research Question 1), followed by an examination of the factors associated with health complaints within these groups (Research Question 2).

### OH faces higher physical and environmental demands than IH, but reports fewer complaints

A central finding of this study is that the IH group reported the highest levels of both musculoskeletal and psychosomatic complaints. The IH group is characterized by the highest work intensity and the most unfavorable working hours, whereas the OH group faces the highest physical and environmental demands. However, regression models reveal that within the IH group, physical and environmental demands remain to have the strongest associations with musculoskeletal complaints, while working time location showed no significant association. It is likely this combination, sustaining physical and environmental demands while simultaneously demonstrating highest work intensity and unfavorable hours, that drives the IH group’s musculoskeletal complaints to the highest levels observed. Furthermore, regarding psychosomatic complaints, work intensity showed to have the strongest associations across all occupational groups. Consequently, the high prevalence of work intensity within the IH group likely explains their elevated psychosomatic complaints, as this detrimental association was most pronounced in this specific group. The IH group appears to face a particularly detrimental constellation of job characteristics. They are exposed to high demands from both physical tasks and intense work pacing and pressure, while simultaneously reporting lower levels of job resources, comparable to those of the OH group. This combination of high demands coupled with low control has been shown to lead to psychological strain [32, 33] and provides robust statistical confirmation of the health impairment process within the JD-R model [15]. An alternative explanation for the slightly lower complaint levels observed in the OH group may be attributable to unmeasured personal or contextual job resources inherent to outdoor work that may buffer the effects of physical and environmental demands [19, 34]. For example, outdoor work might provide greater task variety, more frequent micro-breaks or a sense of freedom that is not captured by the study’s general measure of *autonomy*. Consequently, mitigating employee health complaints in IH occupations may benefit from simultaneously addressing the physical nature of the work and optimizing how that work is structured, particularly regarding its pace, timing and autonomy. This reflects the dual processes of the JD-R model, requiring a focus not only on reducing excessive demands but also on actively building job resources [15].

As expected, employees in the OH group are exposed to the highest levels of physical and environmental demands. This finding empirically validates the concerns raised by international bodies regarding the increased vulnerability of outdoor workers [1]. Additionally, similar to the IH group, OH workers report significantly lower levels of autonomy and social support compared to their low-physical-demand counterparts. The lower level of autonomy in this group is especially alarming in the context of climate change. Autonomy in an outdoor setting is not just a psychosocial comfort, but it is essential for self-protection. It represents the ability to react to environmental threats, for example, by taking a break in the shade during a heatwave, rescheduling the most exhausting tasks to cooler parts of the day or accessing and using protective equipment. The observation that the employees facing the greatest environmental exposure also has the least capacity to manage that exposure, highlights a fundamental weakness in current approaches to workplace health and safety. Empowering outdoor workers with greater control over their work is not just a matter of improving job satisfaction but is an essential occupational health strategy for climate change adaptation [35]. However, it should be taken into account that our dataset reflects conditions in Germany only. Factors such as climate patterns, seasonal variability and occupational norms for outdoor employees can differ between countries and regions. Data from the European Working Conditions Survey 2015 (EWCS) indicate, for example, that only a fifth of German employees are exposed to high temperatures, while in Spain more than a third (36%) are affected [36].

While aggregating occupations into broad groups reveals general trends, these mean values mask substantial heterogeneity. For instance, within the IH group, the occupation of *Elderly Care* (*n* = 64) deviates from the group average, exhibiting higher demands regarding work intensity (4.48 vs. 3.37) and working time location (2.63 vs. 1.40) (see Table 2 in Additional File 1). These higher demands, which align with well-documented stressors specific to the care sector [35], are mirrored by higher rates for psychosomatic (3.98 vs. 3.32) and musculoskeletal complaints (4.05 vs. 2.90). Comparable intra-group differences are evident within the OH group. The *Education and Social Work* sector (*n* = 79) presents as a high-strain outlier, with values for work intensity (3.49 vs. 2.77) and psychosomatic complaints (3.90 vs. 2.71) that far exceed the OH average. Conversely, *Horticulture* (*n* = 38) displays a different profile, with physical demands considerably higher than the group mean (2.95 vs. 2.50) and work intensity exceptionally lower (1.89 vs. 2.77). Furthermore, *Horticulture* workers report minimal demands regarding working time location (0.32), contrasting with both the overall OH average (0.93) and the *Education and Social Work* group (1.21). All specific occupations cited in these examples correspond to the 3-digit level of the revised 2020 version of the Classification of Occupations 2010 (KldB 2010), issued by the Federal Employment Agency (Bundesagentur für Arbeit) [37]. The observed variances suggests that while broad distinctions are useful, occupation-specific context remains crucial for identifying at-risk populations. While this work focused on the effects of work location and varying levels of physical demands in general, Job-Exposure-Matrices (JEMs) can offer a more detailed overview about the stressor-strain profiles of specific occupations [29, 30]. Future research should also explore innovative technologies, such as connected wearable sensors. Such sensor systems can assess physical and environmental demands in real-time [38–40]. They offer the potential to assess the specific stressors of an occupation and the individual physiological reactions of the worker, thereby providing information to derive situation-specific recommendations to mitigate demands effectively.

Consistent with the JD-R model, the OL and IL groups, characterized by lower overall demands alongside higher levels of autonomy and social support, generally reported fewer health complaints than the OH and IH groups.

### Relationship between work conditions and health complaints by occupational group

The regression analyses offer a detailed perspective on the mechanisms linking work conditions to health outcomes. The results, summarized in Figs. 1 and 2, depict the direction, magnitude and significance of each associated variable with musculoskeletal and psychosomatic complaints across the four occupational groups. The findings provide strong support for the core factors of the JD-R model. First, higher work intensity was associated to both musculoskeletal and psychosomatic complaints in all four groups, confirming its role as a consistent job demand that demands employees’ physical and mental resources. Conversely, the job resources of autonomy and social support were consistently associated with fewer complaints across all groups, emphasizing their general importance as protective factors for employee well-being.

An interesting finding emerged regarding the relationship between environmental demands and health complaints within the OH group. In the primary models using dichotomized scales, this relationship appeared non-significant, which contradicted the intuitive expectation that the heaviest environmental exposure should produce strong negative health outcomes. However, our sensitivity analyses utilizing continuous scales revealed that environmental demands are, in fact, significantly associated with both musculoskeletal and psychosomatic complaints in the OH group. This discrepancy highlights how dichotomizing variables can mask true effects by reducing variance. The OH group likely constitutes a self-selected group of resilient individuals with high personal resources who have normalized extreme environmental conditions (e.g., heat, cold, noise) as a baseline part of “the job” [19, 41, 42]. Consequently, these workers may under-report the severity of their exposure, meaning their scores fail to cross a predefined, dichotomized “high risk” threshold. Relying on dichotomized self-reported data may underestimate climate-related demands in highly habituated groups. This reinforces the importance of utilizing continuous scales and supplementing survey-based research with objective measures to fully capture the health impact of environmental exposures.

The regression models identified another paradoxical finding. Within the OH group, working on weekends or outside standard hours (unfavorable working time location) was associated with fewer musculoskeletal complaints. This relationship is unlikely to be directly causal. The most plausible explanation is that, for the OH group, weekend or off-hour work reflects a high-resource work environment. During these periods, employees likely experience reduced supervision, fewer administrative disruptions, less overload and greater flexibility to regulate their work pace. These conditions might collectively represent a form of job resource, an “off-hours autonomy” that enhances perceived control and reduces strain. For instance, surgical residents were found to have greater operative autonomy at night and on weekends, often serving as primary surgeons, suggesting that off-hour work can enhance control and independence [43]. Similarly, night shifts in nursing are often characterized by lower acute job demands; reduced patient contact and observation requirements can afford nurses more resting periods while on duty [44]. Identifying and replicating the specific high-resource characteristics of off-hour work during regular working hours could therefore represent a resource-building intervention consistent with the motivational process of the JD-R model. Notably, no similar relationship emerged for psychosomatic complaints, suggesting that potential negative aspects of off-hour work, such as circadian disruption or social isolation [45, 46] may counterbalance the benefits of increased autonomy. Furthermore, this counterintuitive finding may be largely driven by a healthy worker survivor effect and reverse causation. It is highly plausible that healthier employees, who inherently experience fewer musculoskeletal complaints, disproportionately self-select or are assigned to more demanding, non-standard shifts. Conversely, employees already experiencing health complaints may actively transition to standard working hours as a protective measure.

The findings demonstrate that the JD-R model offers a robust framework. The model’s dual processes are reflected in the associations of demands and resources with health complaints. The health impairment process is evident in the positive association between demands like work intensity and physical demand with negative health outcomes. Simultaneously, the protective and motivational aspects of job resources are confirmed by the consistent negative association between autonomy and social support with the same health outcomes. The model’s sensitivity to context and flexibility in interpretation allow it to account for the unexpected findings of this study. The results observed in the OH group do not contradict the model’s assumptions, but instead broaden its explanatory scope by highlighting that the impact of a working condition depends on the specific combination of factors within a given occupational environment [34] and on the personal resources of the individual or occupational group [19]. This also highlights the necessity of tailored analyses and interventions rather than a one-size-fits-all approach to improving employee well-being.

### Limitations and future directions

The primary strength of this study lies in its use of the BIBB/BAuA Employment Survey 2024, a large, nationally representative dataset. This foundation enhances the generalizability of the findings to the broader German workforce and provides sufficient statistical power for robust analyses. Nevertheless, several limitations warrant consideration. First, the cross-sectional design limits causal interpretation. Second, reliance on self-report measures for all variables introduces the potential for common method variance and reporting biases, including social desirability effects or the influence of negative affectivity on both perceptions of work and reported complaints [47]. Third, while analytically useful, the four occupational groups remain broad and heterogeneous. Fourth, the analyses relied on scales with only limited reliability, which may have led to an underestimation of their associations with other variables. Nonetheless, the Cronbach’s alpha values are comparable to those reported by Meyer and Siefer [29], who used data from the BIBB/BAuA Employment Survey 2018 with similar measurement scales. Fifth, it is important to note that the adjusted R^2^ values in our models indicate that the included job demands and resources explain a modest portion of the overall variance in musculoskeletal and psychosomatic complaints. However, this is expected in occupational health and psychological research. Employee health is a multifactorial phenomenon influenced by a wide array of private-life factors not captured within the JD-R framework. Despite the unmeasured variance, the statistically significant main effects observed remain theoretically and practically meaningful, as they identify specific workplace conditions that organizations can target to improve employee well-being. Another methodological limitation concerns the dichotomization of the job demands and resources variables. Recoding ordinal responses into binary categories can result in a substantial loss of variance and information. Nevertheless, this procedure is commonly adopted in comparable studies [29]. Additionally, interpreting the effect sizes for physical job demands presents a methodological limitation. Because our occupational groups were inherently defined and categorized by their physical intensity, the variance of this variable within the groups is artificially restricted. Consequently, the associations calculated between physical demands and health complaints in our models are likely underestimated. A further methodological limitation involves the use of stratified analyses. Because separate regression models were computed for each occupational group to identify the strongest associations within those specific contexts, it cannot be statistically confirmed whether the magnitude of these associations differs significantly between the groups. Thus, comparisons of effect sizes across groups should be interpreted as descriptive rather than inferential. A final methodological consideration refers to the assessment of health complaints. Because a secondary dataset was utilized, the analysis was restricted to the pre-defined survey items. Consequently, health complaints were assessed using a self-reported sum index rather than a validated scale. Nevertheless, this index has been utilized in previous studies drawing from this national dataset [29, 30].

These limitations suggest several directions for future research. First, panel studies are needed to follow workers over time, strengthening causal interpretation regarding the long-term impact of working conditions on health complaints. Second, mixed-methods approaches that combine large-scale survey data with qualitative interviews could provide insights into the subjective experience of work and help explain some of the paradoxical findings observed here. Third, integrating objective data could complement and validate self-reported measures, for example by linking survey responses to company records of sickness absence and occupational injuries. Finally, our explorative analyses highlighted the highly heterogeneous character of the occupational groups, underscoring the importance of considering occupation-specific contexts when developing interventions.

## Conclusion

This study offers a comprehensive examination of self-reported working conditions and health outcomes in Germany, revealing significant differences across different segments of the workforce. By distinguishing four occupational groups, the analysis demonstrates that employees in predominantly indoor, high-demand (IH) roles report the most musculoskeletal and psychosomatic complaints. While employees in predominantly outdoor, high demand (OH) roles face the strongest environmental and physical demands, they reported less health complaints than employees in the IH group. Furthermore, the absence of a direct association between environmental demands and health complaints within the OH group suggests the influence of unmeasured protective factors, such as personal resources. Consistent with the JD-R model, findings across all occupational groups highlight the direct associations of working conditions with employee health. While work-related demands are consistently linked to increased health complaints, job resources, particularly autonomy and social support, play a beneficial role in promoting overall well-being. However, given the substantial heterogeneity observed within the defined occupational groups, generic interventions may fall short. Therefore, future strategies should also leverage smart, adaptive technologies (e.g., IoT wearables) to deliver situation-specific assistance tailored to the individual occupational context.

## Supplementary Information

Additional file 1. Table 1. Item codes and descriptions from the BIBB/BAuA Employment Survey 2024, categorized by job demands (physical, environmental, intensity, working time), job resources (autonomy, social support), and health outcomes. Scales categorized according to Meyer & Siefer [29, 30]. Descriptions translated from German. Table 2. Descriptive statistics for all dependent variables across specific occupational subgroups. Fig. 1. Regression models examining the associations of job demands and resources with musculoskeletal complaints across four occupational groups (OH, OL, IH, IL) using continuous scales. Values presented are standardized beta coefficients (β) with heteroscedasticity-consistent standard errors (HC3) in parentheses. Bold values indicate significance. All models were adjusted for age, gender, weekly working hours, job tenure, educational attainment, employment status, and job requirement level. Model statistics are provided on the right. Note: the sample sizes (n) differ from the primary models utilizing dichotomized scales due to the omission of item F410 from the work intensity scale, as its response format is not ordinal. Fig. 2. Regression models examining the associations of job demands and resources with musculoskeletal complaints across four occupational groups (OH, OL, IH, IL) using continuous scales. Values presented are standardized beta coefficients (β) with heteroscedasticity-consistent standard errors (HC3) in parentheses. Bold values indicate significance. All models were adjusted for age, gender, weekly working hours, job tenure, educational attainment, employment status, and job requirement level. Model statistics are provided on the right. Note: the sample sizes (n) differ from the primary models utilizing dichotomized scales due to the omission of item F410 from the work intensity scale, as its response format is not ordinal.

## Data Availability

A scientific use file (no. ZA9065) for the BIBB/BAuA 2024 Survey will be available from the first quarter of 2026 via the BIBB Research Data Center (BIBB-FDZ; https://www.bibb.de/de/217152.php).

## Abbreviations

IH: Indoor, High demand
IL: Indoor, Low demand
JD-R: Job Demand-Resources
OH: Outdoor, High demand
OL: Outdoor, Low demand
