# Supplementary material for: Working conditions and state of health of employees working indoor and outdoor with varying levels of physical demand: Insights from the BIBB/BAuA employment survey 2024
Source: BMC Public Health. 2026 Jun 11;26:1859. doi: 10.1186/s12889-026-28055-z (PMC13262516; doi:10.1186/s12889-026-28055-z)
Supplement: Supplementary file 1 — Additional file 1. Table 1. Item codes and descriptions from the BIBB/BAuA Employment Survey 2024, categorized by job demands (physical, environmental, intensity, working time), job resources (autonomy, social support), and health outcomes. Scales categorized according to Meyer & Siefer [29, 30]. Descriptions translated from German. Table 2. Descriptive statistics for all dependent variables across specific occupational subgroups. Fig. 1. Regression models examining the associations of job demands and resources with musculoskeletal complaints across four occupational groups (OH, OL, IH, IL) using continuous scales. Values presented are standardized beta coefficients (β) with heteroscedasticity-consistent standard errors (HC3) in parentheses. Bold values indicate significance. All models were adjusted for age, gender, weekly working hours, job tenure, educational attainment, employment status, and job requirement level. Model statistics are provided on the right. Note: the sample sizes (n) differ from the primary models utilizing dichotomized scales due to the omission of item F410 from the work intensity scale, as its response format is not ordinal. Fig. 2. Regression models examining the associations of job demands and resources with musculoskeletal complaints across four occupational groups (OH, OL, IH, IL) using continuous scales. Values presented are standardized beta coefficients (β) with heteroscedasticity-consistent standard errors (HC3) in parentheses. Bold values indicate significance. All models were adjusted for age, gender, weekly working hours, job tenure, educational attainment, employment status, and job requirement level. Model statistics are provided on the right. Note: the sample sizes (n) differ from the primary models utilizing dichotomized scales due to the omission of item F410 from the work intensity scale, as its response format is not ordinal. [file 12889_2026_28055_MOESM1_ESM.pdf]

Additional file 1

Article title: Working Conditions and State of Health of Employees Working Indoor and Outdoor with Varying Levels of Physical Demand: Insights from the 2024 BIBB/BAuA Employment Survey
Journal name: BMC Public Health
Author names: Norman Riedel, Sarah Steghaus, Sascha Wischniewski
Affiliation: Federal Institute for Occupational Safety and Health, Dortmund, Germany
E-Mail of corresponding author: riedel.norman@baua.bund.de

Table 1 Item codes and descriptions from the 2024 BIBB/BAuA Employment Survey, categorized by job demands (physical, environmental, intensity, working time), job resources (autonomy, social support), and health outcomes. Scales categorized according to Meyer & Siefer [29, 30]. Descriptions translated from German

Item	Description
**Physical demands (4 Items)**	
F600_01	Working while standing.
F600_03	Lifting and carrying loads weighing more than 20 kg for male; 10 kg for female.
F600_07a	Performing tasks with your hands that require a high degree of dexterity, rapid sequences of movements, or considerable strength.
F600_07d	Working in a squatting or kneeling position.
**Environmental demands (6 Items)**	
F600_04	Working in smoke, dust, or around gases or vapors.
F600_05	Working in cold, heat, wet conditions, humidity, or drafts.
F600_06	Working with oil, grease, dirt, grime.
F600_12	Working in noisy conditions.
F600_12a	Working amid distracting noises.
F600_13	Dealing with infectious agents, e.g., bacteria or viruses.
**Work intensity (7 Items)**	
F410	Do you generally feel up to the demands of the workload or the amount of work, or do you feel rather overwhelmed or rather underchallenged?
F411_01	Work under intense deadline or performance pressure.
F411_06	Being disturbed or interrupted at work, e.g., by coworkers, poor materials, machine malfunctions, or phone calls.
F411_09	Keep track of different tasks or processes simultaneously.
F411_12	Push yourself to the limits of your abilities.
F411_13	Work very quickly.

Item	Description
F411_16	Unable to cope with the amount of information generated by your work.
**Working time location (3 Items)**	
F220	Do you usually work on Saturdays at least once a month?
F223	Do you usually work on Sundays at least once a month?
F209	Are your working hours usually between 7 a.m. and 7 p.m.?
**Autonomy (6 Items)**	
F700_02	Being able to plan and organize one's own work.
F700_03	Influence on the assigned workload.
F700_05	Having a say in what is to be achieved at work.
F700_06	Being able to decide for yourself when to take a break.
F411_18	You are solely responsible for checking your work results.
F411_19	Have to decide for yourself how the work is done.
**Social support (5 Items)**	
F700_10	Feeling part of a community at work.
F700_11	How often do you feel that the cooperation between you and your colleagues is good?
F700_12	How often do you receive help and support for your work from col-leagues when you need it?
F700_13	How often do you receive help and support for your work from your immediate supervisor when you need it?
F700_14	How often does your immediate supervisor praise and recognize you when you do a good job?
**Musculoskeletal complaints (8 Items)**	
F1500_01	Lower back pain, lumbar pain
F1500_02	Pain in the neck and shoulder area
F1500_03a	Pain in the arms
F1500_03b	Pain in the hands
F1500_04	Pain in the hip
F1500_05	Pain in the knees
F1500_06a	Swollen legs
F1500_06b	Pain in the legs, feet
**Psychosomatic complaints (11 Items)**	
F1500_07	Head ache
F1500_08	Heart pain, stabbing pains, pain, or tightness in the chest

Item	Description
F1500_09	Shortness of breath
F1500_13	Skin irritation, itching
F1500_14	Nighttime sleep disturbances
F1500_15	General tiredness, fatigue, or exhaustion
F1500_16	Stomach or digestive problems
F1500_17	Hearing loss, tinnitus
F1500_18	Nervousness or irritability
F1500_19	Depression
F1500_20	Dizziness

Table 2 Descriptive statistics for all dependent variables across specific occupational sub-groups

Variables	**Elderly care** (within IH group)		**Education and Social Work** (within OH group)		**Horticulture** (within OH group)	
	M (*SD*)	*n*	*M* (*SD*)	*n*	*M* (*SD*)	*n*
**Demands**						
Physical Demands	3.22 (0.79)	63	2.47 (1.15)	78	2.95 (1.01)	38
Environmental Demands	2.03 (1.21)	64	2.81 (1.17)	79	2.76 (1.38)	38
Work Intensity	4.48 (1.55)	63	3.49 (1.79)	79	1.89 (1.94)	38
Working Time Location	2.63 (0.58)	64	0.39 (0.79)	79	0.32 (0.53)	38
**Resources**						
Autonomy	3.37 (1.58)	63	3.56 (1.36)	78	3.45 (1.74)	38
Social Support	2.94 (1.64)	64	3.68 (1.35)	74	3.47 (1.23)	36
**Complaints**						
Musculoskeletal Complaints	4.05 (1.96)	64	3.22 (2.29)	79	3.03 (2.21)	38
Psychosomatic Complaints	3.98 (2.39)	64	3.90 (2.69)	79	2.30 (1.87)	37

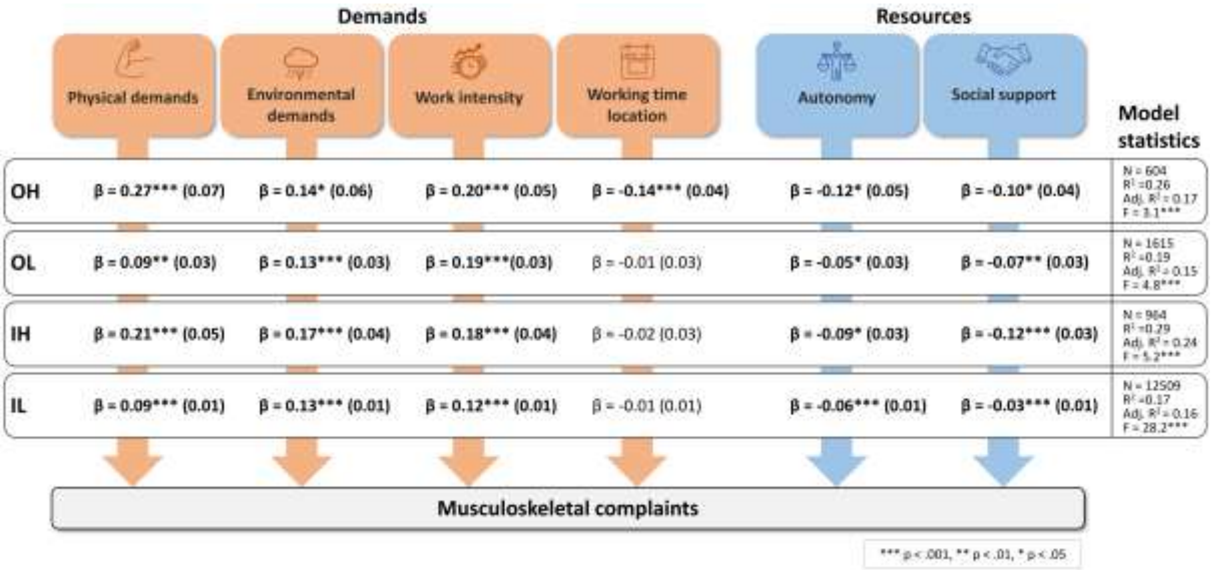

Fig. 1 Regression models examining the associations of job demands and resources with musculoskeletal complaints across four occupational groups (OH, OL, IH, IL) using continuous scales. Values presented are standardized beta coefficients (β) with heteroscedasticity-consistent standard errors (HC3) in parentheses. Bold values indicate significance. All models were adjusted for age, gender, weekly working hours, job tenure, educational attainment, employment status, and job requirement level. Model statistics are provided on the right. *Note:* the sample sizes (n) differ from the primary models utilizing dichotomized scales due to the omission of item F410 from the work intensity scale, as its response format is not ordinal

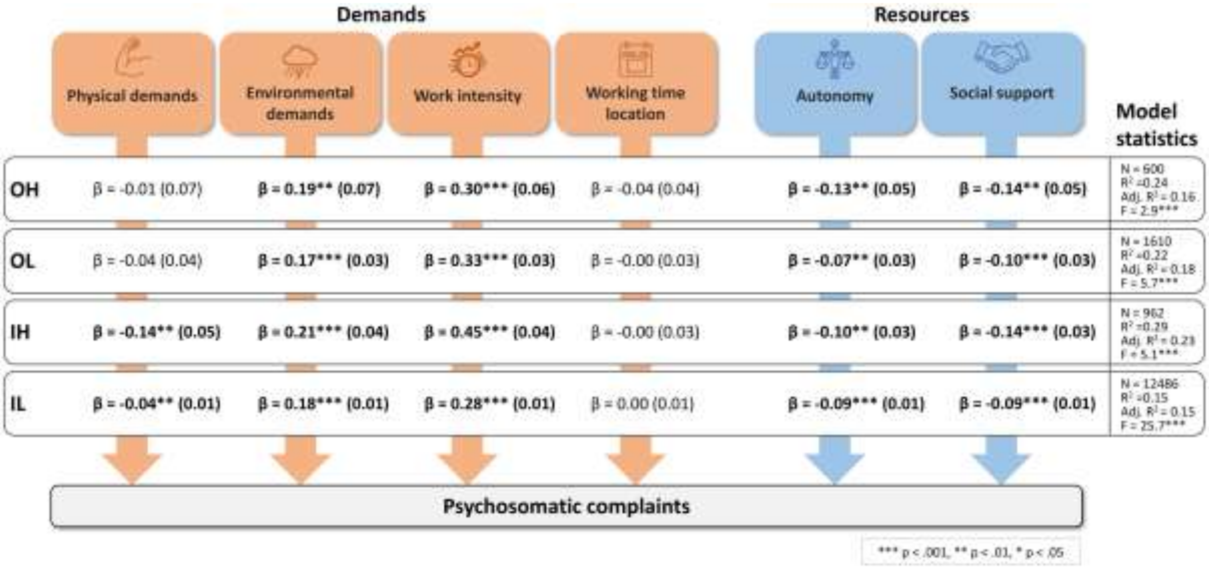

Fig. 2 Regression models examining the associations of job demands and resources with musculoskeletal complaints across four occupational groups (OH, OL, IH, IL) using continuous scales. Values presented are standardized beta coefficients (β) with heteroscedasticity-consistent standard errors (HC3) in parentheses. Bold values indicate significance. All models were adjusted for age, gender, weekly working hours, job tenure, educational attainment, employment status, and job requirement level. Model statistics are provided on the right. *Note:* the sample sizes (n) differ from the primary models utilizing dichotomized scales due to the omission of item F410 from the work intensity scale, as its response format is not ordinal
